# Nutritional Adequacy of Dietary Intake in Women with Anorexia Nervosa

**DOI:** 10.3390/nu7053652

**Published:** 2015-05-15

**Authors:** Susan K. Raatz, Lisa Jahns, LuAnn K. Johnson, Ross Crosby, James E. Mitchell, Scott Crow, Carol Peterson, Daniel Le Grange, Stephen A. Wonderlich

**Affiliations:** 1 USDA, ARS, Human Nutrition Research Center, Grand Forks, ND 58203, USA; E-Mails: lisa.jahns@ars.usda.gov (L.J.); luann.johnson@ars.usda.gov (L.K.J.); 2 Department of Food Science and Nutrition, University of Minnesota, Saint Paul, MN 55108, USA; 3 Neuropsychiatric Research Institute, Fargo, ND 58103, USA; E-Mails: Rcrosby@nrifargo.com (R.C.); Jmitchell@nrifargo.com (J.E.M.); SWonderlich@nrifargo.com (S.A.W.); 4 Department of Clinical Neurosciences, University of North Dakota, Grand Forks, ND 58202, USA; 5 Department of Psychiatry, University of Minnesota Medical School, Minneapolis, MN 55455, USA; E-Mails: crowx002@umn.edu (S.C.); peter161@umn.edu (C.P.); 6 Department of Psychiatry and Department of Pediatrics, University of California, San Francisco, San Francisco, CA 94143, USA; E-Mail: Daniel.LeGrange@ucsf.edu

**Keywords:** anorexia nervosa, dietary intake, WWEIA, NHANES 2011–2012

## Abstract

Understanding nutrient intake of anorexia nervosa (AN) patients is essential for the treatment. Therefore, estimates of total energy and nutrient consumption were made in a group of young women (19 to 30 years) with restricting and binge purge subtypes of AN participating in an ecological momentary assessment study. Participants completed three nonconsecutive 24-hour diet recalls. Mean nutrient intakes were stratified by subtype and by quartiles of energy intake and compared to the age specific Dietary Reference Intake (DRI) levels, as well as to the reported intakes from the What We Eat In America (WWEIA) dietary survey 2011–2012. Reported intake was determined for energy, macronutrients, and micronutrients. The mean body mass index (BMI) for all participants was 17.2 ± 0.1 kg/m^2^. Reported nutrient intake was insufficient for participants in quartiles 1–3 of both AN subtypes when compared to the DRIs. Intake reported by participants in quartile 4 of both subgroups met requirements for most nutrients and even met or exceeded estimated energy needs. Counseling of AN patients should be directed to total food consumption to improve energy intake and to reduce individual nutritional gaps.

## 1. Introduction

Anorexia nervosa (AN) is a psychiatric disorder that results in considerable morbidity and mortality. The estimated prevalence is between 0.3% and 0.6% in adolescents and young adult women [1]. Prolonged dietary restriction in this disorder results in overt malnutrition, leading to significant decline in health status. In addition to the obvious wasting and loss of lean and fat mass, patients experience bone loss and often amenorrhea [2]. Onset of AN at an early age leads to reduced growth and an inability to attain genetic height potential despite accelerated growth following nutritional therapy [3]. Long-term mortality associated with the disease is ~10% per decade mainly due to cachexia and suicide [1,4]. 

AN is characterized by two subtypes-those with primarily food restricting behavior and those with associated binge eating and purging behavior [5]. Although definitions of the subtypes are quite distinct, frequent crossover can occur, with up to 62% of restricting patients developing binge purge behavior [6]. Knowledge of the AN subtype is important for selection of therapeutic approaches to treatment, and may play an important role in the nutritional and medical status of the individual [7].

Understanding the nutrient intake of AN patients is essential for nutritional counseling and treatment. Assessment of dietary intake in this population is fraught with error [8]. Studies of dietary consumption with retrospective recall of intake have obvious shortcomings as individuals with AN are often less than candid about their eating behavior; however, their knowledge of what was consumed may be greater than that of the general population [9]. Obtaining dietary intake information by observation in a hospital or clinic setting is not representative of usual intake; therefore, dietary recall is the best available technique to assess consumption and has high agreement between reported total energy intake and predicted energy expenditure [10].

Recently the Academy of Nutrition and Dietetics published its Position Paper on *Nutrition Intervention in the Treatment of Eating Disorders* [11]. This statement contends that “nutrition intervention, including nutrition counseling by a registered dietitian, is an essential component of the team treatment of patients with anorexia nervosa, bulimia nervosa, and other eating disorders during assessment and treatment across the continuum of care” [11]. Information on adequacy of nutrient intake in this group will direct clinically relevant medical nutrition therapy emphasizing common key nutritional deficiencies in the context of a treatment plan for a healthy and nutritionally complete diet.

In evaluation of dietary intake of individuals with AN, emphasis has been primarily given to the reduced intake of energy and macronutrients. Little work has been done to describe the effect of diet restriction on the total micronutrient intake levels in AN [10,12,13]. Our objective was to quantify nutrient intake in a group of participants with AN who were taking part in an ecological momentary assessment study (EMA). Therefore, we assessed total reported energy and nutrient intake in a sample of young women, aged 19 to 30 years, with both restricting and binge purge subtypes of AN and compared them to the Dietary Reference Intakes (DRIs) [14] for women aged 19–30 years. We also compared this group to the reported intake of a representative group of women who participated in What We Eat in America (WWEIA) the dietary intake segment of the of the National Health and Nutrition Examination Survey (NHANES) 2011–2012 [15,16]. We discuss the relevance of our results to RDs and other practitioners working with AN patients.

## 2. Experimental Section

### 2.1. Experimental Protocol

This study is an analysis of dietary recall data that was part of an EMA study in which participants with AN provided information on eating behavior, emotions, stress and coping on hand held computers over 14 days [17]. Participants also completed three non-consecutive 24-hours dietary recalls (DR) during the two weeks. The study was conducted between 2006 and 2010. The main outcome, adequacy of energy and nutrient intakes, is compared to the DRI and intake of participants in WWEIA/NHANES 2011–2012. Studies were carried out at the Neuropsychiatric Institute, Fargo, ND; The University of Minnesota, Minneapolis, MN and The University of Chicago, Chicago, IL. Approval for this protocol was obtained from Institutional Review Boards of all facilities involved in this research: The University of North Dakota, Grand Forks, ND; Sanford Health, Fargo, ND; the University of Minnesota; and the University of Chicago. All participants provided written informed consent prior to enrollment in the study.

### 2.2. Participants

Participants in the trial included women aged 17 to 58 years (*n* = 118) with a diagnosis of AN. volunteers were recruited to meet the criteria for AN, either restricting or binge purge type, as defined in the Diagnostic and Statistical Manual of Mental Disorders [5] or subclinical AN as previously described [17]. 

A subset of all recruited participants was included in this analysis, who were between the ages of 19 and 30 years, and had three complete days of random DR (*n* = 75; 64% of sample). This age group was used for this assessment as it corresponds to the life stage and sex grouping of the DRI for the purpose of comparison of nutritional adequacy. There were not enough women in the other age groups (14–18, 31–50, or 51–70 years) to provide appropriate aggregate intake data for comparison to the DRIs.

### 2.3. Diagnostic Assessment

Participants were assessed with structured interviews at baseline to evaluate the status of their disorder and to classify their subtype of AN, including the Structured Clinical Interview for DSM IV, Axis I [18] and the Eating Disorders Examination [19]. Body Mass Index (BMI) (kg/m^2^) of participants was determined by measurement of height in cm and weight in kg by trained clinic staff. 

### 2.4. Dietary Assessment

Participants were called three times to obtain detailed 24-hours dietary recall information. The recalls were performed by trained interviewers and detailed diet information was directly entered during the interview and analyzed utilizing the 2005 version of the Nutrition Data System for Research (NDS-R) [20,21] from the Nutrition Coordinating Center at the University of Minnesota. The multiple-pass methodology used by trained interviewers during recalls is similar to the methodology used to collect 24-hours DR data in the WWEIA/NHANES survey [22].

### 2.5. Comparison to Dietary Reference Intakes

Use of DRIs as part of the nutrition care process was discussed in detail in a recent practice dietetics paper [23]. Given the restrictive eating habits of individuals with AN, many nutrient shortfalls in the diet are expected. Individual nutrition assessments include many indicators of nutritional status and often include dietary intake measured by 24-hours DR or food records. If dietary intake has been assessed for ≥3 days, nutrient intake can be compared to the DRIs and included in the treatment process [23]. Current dietary intake data can be used to identify nutrient intakes that are relevant for planning treatment and follow-up goals [24].

The Estimated Average Requirement (EAR) is the median usual intake estimated to meet the physiological needs of half of the individuals in a life stage and sex group. The recommended dietary allowance (RDA) is sufficient to meet or exceed the estimated needs of 98% of the population of interest and is set at two standard deviations above the EAR. An Adequate Intake (AI) is set when there is insufficient scientific evidence to set an EAR and RDA and is the estimated intake of an apparently healthy population. 

The Practice Paper of the American Dietetic Association: *Using the Dietary Reference Intakes* provides guidelines for the application of the DRIs to patient assessment: (1) if the reported intake of a specific nutrient exceeds the RDA or AI, intake may be considered adequate; (2) if intake is less than the AI, it cannot be considered inadequate, but the dietitian may choose to encourage increased consumption to meet the AI; (3) if intake is below the RDA but exceeds the EAR, there is a 50% chance that intake does not meet the individual requirement and increased consumption should be encouraged; (4) if intake is below the EAR there is a greater chance that intake is not sufficient to meet requirements [23].

Estimated Energy Requirement (EER) is defined as “the average dietary intake that is predicted to maintain energy balance in a healthy adult of a defined age, gender, weight, height, and level of physical activity consistent with good health” [25]. The EER does not address energy needs of AN patients, as additional energy requirements to restore healthy weight depends upon factors such as initial deficit, desired rate of recovery, and catch-up growth. Therefore, the EER may underestimate the energy requirements of patients with AN, despite their low body weight. On the other hand, AN patients are hypometabolic and the EER may overestimate their maintenance energy needs if they are not physically active [2]. Due to the uncertainty of AN patients energy needs, the description of EER was calculated and presented at moderate physical activity levels [25].

EER = 354 – 6.91 × age [y] + PA × (9.36 × weight [kg] + 726 × height [m])
(1)

where PA is 1.27 if moderately active.

### 2.6. Comparison to NHANES

For comparison to an apparently healthy population, the nutrient intake of non-pregnant women aged 19–30 years was derived from the 2011–2012 WWEIA/NHANES survey [16]. WWEIA/NHANES is a nationally representative, cross-sectional survey of the non-institutionalized, civilian U.S. population. The survey uses a complex, stratified, multistage probability cluster sampling design. WWEIA/HANES participants were asked to complete a single in-person 24-hours DR. A second 24-hours DR was collected via telephone within 10 days of the first; however, only the day 1 recalls are used in our analysis (*n* = 403 women) [26,27,28].

### 2.7. Statistical Analysis

Nutrient values computed from the three DR were averaged for each subject and divided into quartiles based on reported energy intake within restricting and binge purge subtypes of AN. Means and 95% confidence intervals (CI) were calculated for each nutrient within each quartile. The percentage of women whose reported intakes were less than the EAR or AI, as appropriate, were calculated for each nutrient. Mean and 95% CI of nutrient intakes of women ages 19–30 who participated in WWEIA/NHANES 2011–2012 were calculated using the Survey means procedure in SAS (SAS Institute, Cary, NC). Analyses included the sampling strata and primary sampling units and were weighted using the appropriate NHANES sample weights. Consonance of the AN subjects intakes with those of the nationally representative sample of women aged 19–30 was estimated by calculating the percentage consumed by an AN subject relative to the mean intakes reported by the WWEIA/NHANES respondents. 

Age and BMI values for all women and for AN subgroup by quartile were calculated and presented as mean ± standard error of the mean (SEM). Mean ± SEM of the EER was calculated for each quartile for each AN subgroup. The percentage of subject consuming at least 100% of the EER was calculated for each of the 3 activity levels. All analyses were performed with SAS V9.3 (SAS Institute, Inc., Cary, NC, USA). 

## 3. Results

The overall mean age of participants was 22.5 ± 0.4 years and the average BMI was 17.2 ± 0.1 kg/m^2^. There were no significant differences in age and BMI between the diagnostic subtypes across the quartiles. Age in years (mean ± SEM) and BMI (mean ± SEM) for participants by AN subtype of restricting and binge purge were, respectively Quartile 1: age: 23 ± 1, 20 ± 1, BMI: 17.3 ± 0.3; 17.3 ± 0.3; Quartile 2: age: 22 ± 1, 25 ± 1, BMI: 17.8 ± 0.2; 17.2 ± 0.6, Quartile 3: age: 22 ± 1, 25 ± 1. BMI: 17.0 ± 0.3, 17.3 ± 0.3; Quartile 4: age: 21 ± 1, 24 ± 1.3, BMI: 17.0 ± 0.3; 17.0 ± 0.5.

Table 1 presents mean and 95% CI for reported dietary intake of participants by AN subtype (quartiles) in reference to the DRI. For all nutrients under consideration the percentage of individuals reporting intakes lower than the EAR are shown. Participants of both subtypes in quartiles 1 and 2 reported low energy intake and consequently were below the DRI EAR for those nutrients evaluated. As reported energy intake increased in both AN subtype groups in quartiles 3 and 4, the number of participants not reporting intakes below the EAR is reduced the only macronutrient with an EAR is carbohydrate. The DRIs also suggest evaluating diets based upon the Acceptable Macronutrient Distribution Range (AMDR). These ranges are based upon energy intake. The AMDR for fat, protein and carbohydrate is 20%–25% en, 10%–35% en and 45%–65% en, respectively. However, with the wide variability in reported energy intake in this population it may be more clinically relevant to use the AMDRs to plan diets based upon on estimated energy needs, not reported intake.

Table 2 provides mean reported dietary intake by AN subtype (quartiles) compared to mean reported intake by WWEIA/NHANES for women of the same age range. Reported intakes are substantially lower for all subjects except those in the 4th quartile of both the restrictor and binge purge subtype of AN. Estimated energy requirements for participants at a moderate activity level are 2242 ± 27 kcal, 2292 ± 32 kcal, 2281 ± 28 kcal, 2313 ± 32 kcal, for quartiles 1 through 4, respectively, of the AN restrictor subtype and 2253 ± 25 kcal, 2248 ± 48 kcal, 2355 ± 31 kcal, 2304 ± 37 kcal for quartiles 1–4, respectively, for the AN binge-purge subtype participants. Participants in quartiles 1–3 of both subtypes of AN have very low concordance with energy needs at moderate activity levels with none of the participants reporting intake equivalent to EER. Sixty four percent (64%) of participants in quartile 4 of the AN restrictor subtype and 86% of those in quartile 4 of the AN binge purge subtype meet the EER. 

## 4. Discussion

The mean BMI for all participants (17.2 ± 0.1 kg/m^2^) was, as expected, below the lower limit of normal for healthy individuals. Reported nutrient intake was insufficient for participants in quartiles 1–3 of both AN subtypes when compared with DRIs. Intake reported by quartile 4 of both subgroups met or exceeded the requirements for most nutrients. Reported energy intake ranges from extremely low (~25%–40% of estimated needs) in quartile 1 of both AN subgroups to very high (158%–220% of estimated needs) for participants in quartile 4 of the binge purge AN subtype. Interestingly, when compared to the national sample, participants of both subtypes falling in quartiles 3 and 4 reported similar or higher energy and nutrient intakes. The higher reported intakes may in part be due to over-reporting by the AN population, or reflective of the increased food intake found among individuals engaging in binge-purge behaviors.

**Table 1:** Reported dietary intake of individuals with anorexia nervosa (by subtype and quartile) and percent not meeting the Dietary Reference Intakes.

		Quartile 1				Quartile 2				Quartile 3				Quartile 4			
		Restrictor (*n* = 12)		Binge/Purge (*n* = 8)		Restrictor (*n* = 11)		Binge / Purge (*n* = 7)		Restrictor (*n* = 12)		Binge / Purge (*n* = 7)		Restrictor (*n* = 11)		Binge / Purge (*n* = 7)	
	DRI^1^ *EAR **AI	Mean (95% CI)	% Below EAR	Mean (95% CI)	% Below EAR	Mean (95% CI)	% Below EAR	Mean (95% CI)	% Below EAR	Mean (95% CI)	% Below EAR	Mean (95% CI)	% Below EAR	Mean (95% CI)	% Below EAR	Mean (95% CI)	% Below EAR
Energy (kcal)		736 (567–904)		671 (571–771)		1308 (1194–1421)		1082 (965–1200)		1799 (1706–1892)		1662 (1445–1880)		2639 (2130–3147)		4102 (2301–5904)	
*Carbohydrate (g)	100	110 (81–138)	50	128 (104–151)	25	203 (175–230)	0	161 (122–200)	0	252 (233–270)	0	238 (208–267)	0	382 (316–448)	0	533 (278–788)	0
Protein (g)		33 (22–44)		22 (15–29)		54 (45–63)		41 (25–56)		78 (67–88)		59 (39–80)		93 (76–110)		143 (65–221)	
Fat (g)		17 (13–23)		17 (7.6–16)		35 (30–40)		33 (23–43)		54 (46–63)		57 (46–68)		84 (58–110)		159 (78–240)	
**Dietary Fiber (g)	25	12 (6.4–17)		12 (7–18)		19 (14–23)		12 (0.2–24)		21 (14–28)		17 (10–24)		27 (21–33)		31 (19–43)	
*Calcium (mg)	800	545 (291–799)	75	451 (281–621)	88	761 (578–944)	55	676 (306–1046)	57	1073 (829–1317)	25	926 (413–1440)	57	1514 (1191–1838)	9	2271 (769–3772)	0
*Iron (mg)	8.1	7.9 (4.1–12)	67	9 (2.9–16)	63	15 (10–19)	9	10 (6–13)	43	18 (13–22)	0	15 (8–23)	29	24 (17–31)	0	28 (15–40)	0
*Phosphorus (mg)	580	607 (403–811)	42	453 (308–597)	63	962 (814–1109)	0.0	812.7 (470–1156)	29	1355.6 (1156–1556)	0	1063 (714–1413)	0	1745 (1453–2037)	0	2815 (1052–4578)	0
*Thiamin (mg)	0.9	0.7 (0.4–0.9)	67	1 (0.4–1.7)	75	1 (1.1–1.6)	0.0	1.0 (0.7–1.3)	43	1.7 (1.5–2.0)	0	1.5 (1.1–1.9)	14	2.6 (1.9–3.4)	0	3.43 (1.7–5.2)	0
*Riboflavin (mg)	0.9	1.1 (0.8–1.5)	25	2 (0.9–2.0)	0.0	2 (1.4–2.0)	0.0	2.6 (0.4–4.7)	14	2.4 (2.1–2.8)	0	2.1 (1.3–2.8)	0	3.8 (2.7–4.9)	0	5.3 (1.9–8.8)	0
*Niacin	11	8.6 (5.5–12)	67	13 (4.8–21)	75	19 (14–23)	9	11.6 (8.3–15)	29	23.3 (19–28)	0	18.5 (13–24)	0	35.0 (25–45)	0	43.9 (22–665)	0
*Folate (µg)	320	234 (145–324)	75	291 (158–423)	63	458 (316–601)	18	235 (115–355)	71	497 (341–654)	25	434 (228–640)	43	690 (499–882)	9	764 (343–1185)	14
*Vitamin B6 (mg)	1.1	0.9 (0.6–1.3)	67	1 (0.2–1.8)	88	2 (1.2–1.8)	27	1.0 (0.5–1.4)	71	2.1 (1.3–2.9)	8	1.6 (0.8–2.4)	43	3.1 (2.3–4.0)	0	3.6 (1.4–5.8)	0
*Vitamin B12 (µg)	2	2.2 (1.0–3.4)	58	2 (0.3–4.3)	63	3 (2.0–3.9)	18	3.2 (1.3–5.0)	43	5.8 (3.9–8.0)	0	4.8 (2.0–7.6)	29	8.4 (5.8–11.0)	0	11.7 (1.6–22)	0
*Vitamin C (mg)	60	70 (38–102)	50	85 (25–146)	50	77 (37–117)	55	44 (2–87)	71	116 (76–157)	17	96 (39–153)	14	129 (85–174)	27	107 (54–160)	29
*Vitamin A(µg RE)	500	503 (286–720)	50	536 (110–962)	50	850 (580–1120)	18	567 (229–905)	57	811 (543–1078)	33	916 (374–1457)	29	1235 (887–1584)	9	2421 (449–4394)	0
*Vitamin D (µg)	10	1.7 (0.5–2.9)	100	2 (0.6–2.4)	100	3 (2.4–4.5)	100	2.5 (0.5–4.6)	100	6.0 (3.2–8.9)	92	4.3 (1.6–7.0)	100	6.7 (4.6–8.8)	91	14.7 (3.9–26)	43
*Vitamin E (mg)	12	2.7 (1.5–3.9)	100	5 (0.3–9.9)	88	6 (4.0–7.0)	100	4.4 (2.2–6.5)	100	8.7 (5.0–12)	67	6.2 (4.2–8.2)	100	16.2 (9.6–23)	64	12.2 (8.8–16)	57
**Vitamin K (µg)	90	88 (34–142)		112 (7–217)		112 (47–178)		68 (19–117)		90 (49–131)		107 (35–247)		139 (69–208)		143 (75–211)	
*Selenium (µg)	45	39 (27–52	67	37 (17–56)	88	76 (65–88)	9	59 (37–80)	43	107 (88–126)	0	83 (47–119)	14	125 (104–146)	0	202 (89–314)	0
*Zinc (mg)	6.8	5.4 (3.4–7.3)	67	7 (2.6–5.7)	88	9 (6.4–12)	27	5.6 (3.0–8.2)	86	10.6 (9.6–12)	0	9.6 (5.3–14)	29	16.3 (12–21)	0	19.9 (7.2–33)	0

^1^ DRI, Dietary Reference Intakes; * EAR, Estimated Average Requirement; ** AI, Adequate Intake.

**Table 2:** Reported dietary intake of individuals with anorexia nervosa (by subtype and quartile) and comparison (%) to a representative group from NHANES 2011–2012

		Quartile 1				Quartile 2				Quartile 3				Quartile 4			
		Restrictor (*n* = 12)		Binge/Purge (*n* = 8)		Restrictor (*n* = 11)		Binge/Purge (*n* = 7)		Restrictor (*n* = 12)		Binge/Purge (*n* = 7)		Restrictor (*n* = 11)		Binge/Purge (*n* = 7)	
	**NHANES**	Mean (95% CI)	%	Mean (95% CI)	%	Mean (95% CI)	%	Mean (95% CI)	%	Mean (95% CI)	%	Mean (95% CI)	%	Mean (95% CI)	%	Mean (95% CI)	%
Energy (kcal)	2003 (1913–2094)	736 (567–904)	37	671 (571–771)	34	1308 (1194–1421)	65	1082 (965–1200)	54	1799 (1706–1892)	90	1662 (1445–1880)	83	2639 (2130–3147)	132	4102 (2301–5904)	205
Carbohydrate (g)	252 (239–264)	110 (81–138)	44	128 (104–151)	51	203 (175–230)	80	161 (122–200)	64	252 (233–270)	100	238 (208–267)	95	382 (316–448)	152	533 (278–788)	212
Protein (g)	72 (68–75)	33 (22–44)	46	22 (15–29)	31	54 (45–63)	75	41 (25–56)	57	78 (67–88)	108	59 (39–80)	83	93 (76–110)	129	143 (65–221)	199
Fat (g)	75 (71–79)	17 (13–23)	23	17 (7.6–16)	15	35 (30–40)	47	33 (23–43)	44	54 (46–63)	73	57 (46–68)	76	84 (58–110)	112	159 (78–240)	213
*Dietary Fiber (g)	16 (14–17)	12 (6.4–17)	73	12 (7–18)	78	19 (14–23)	118	12 (0.2–24)	76	21 (14–28)	134	17 (10–24)	108	27 (21–33)	171	31 (19–43)	199
Calcium (mg)	909 (835–983)	545 (291–799)	60	451 (281–621)	50	761 (578–944)	84	676 (306–1046)	74	1073 (829–1317)	118	926 (413–1440)	102	1514 (1191–1838)	167	2271 (769–3772)	250
Iron (mg)	14 (13–15)	7.9 (4.1–12)	57	9 (2.9–16)	68	15 (10–19)	106	10 (6–13)	68	18 (13–22)	127	15 (8–23)	110	24 (17–31)	171	28 (15–40)	197
Phosphorus (mg)	1229 (1168–1290)	607 (403–811)	49	453 (308–597)	37	962 (814–1109)	78	812.7 (470–1156)	66	1355.6 (1156–1556)	110	1063 (714–1413)	87	1745 (1453–2037)	142	2815 (1052–4578)	229
Thiamin (mg)	1.6 (1.4–1.7)	0.7 (0.4–0.9)	44	1 (0.4–1.7)	66	1 (1.1–1.6)	89	1.0 (0.7–1.3)	63	1.7 (1.5–2.0)	109	1.5 (1.1–1.9)	97	2.6 (1.9–3.4)	166	3.43 (1.7–5.2)	219
Riboflavin (mg)	1.8 (1.7–2.0)	1.1 (0.8–1.5)	61	2 (0.9–2.0)	79	2 (1.4–2.0)	93	2.6 (0.4–4.7)	138	2.4 (2.1–2.8)	131	2.1 (1.3–2.8)	112	3.8 (2.7–4.9)	206	5.3 (1.9–8.8)	287
Niacin	23 (22–25)	8.6 (5.5–12)	37	13 (4.8–21)	56	19 (14–23)	80	11.6 (8.3–15)	50	23.3 (19–28)	101	18.5 (13–24)	80	35.0 (25–45)	152	43.9 (22–665)	190
Folate (µg)	390 (349–430)	234 (145–324)	60	291 (158–423)	75	458 (316–601)	118	235 (115–355)	60	497 (341–654)	128	434 (228–640)	111	690 (499–882)	177	764 (343–1185)	196
Vitamin B6 (mg)	1.9 (1.8–2.1)	0.9 (0.6–1.3)	47	1 (0.2–1.8)	51	2 (1.2–1.8)	77	1.0 (0.5–1.4)	50	2.1 (1.3–2.9)	107	1.6 (0.8–2.4)	84	3.1 (2.3–4.0)	163	3.6 (1.4–5.8)	186
Vitamin B12 (µg)	4.5 (4.0–4.9)	2.2 (1–3.4)	48	2 (0.3–4.3)	52	3 (2.0–3.9)	66	3.2 (1.3–5.0)	71	5.8 (3.9–8.0)	130	4.8 (2.0–7.6)	107	8.4 (5.8–11.0)	188	11.7 (1.6–22)	261
Vitamin C (mg)	83 (69–96)	70 (38–102)	85	85 (25–146)	103	77 (37–117)	93	44 (2–87)	54	116 (76–157)	141	96 (39–153)	117	129 (85–174)	156	107 (54–160)	130
Vitamin A (µg RE)	595 (511–679)	503 (286–720)	85	536 (110–962)	90	850 (580–1120)	143	567 (229–905)	95	811 (543–1078)	136	916 (374–1457)	154	1235 (887–1584)	208	2421 (449–4394)	407
Vitamin D (µg)	3.7 (3.1–4.3)	1.7 (0.5–2.9)	47	2 (0.6–2.4)	40	3 (2.4–4.5)	92	2.5 (0.5–4.6)	68	6.0 (3.2–8.9)	163	4.3 (1.6–7.0)	116	6.7 (4.6–8.8)	181	14.7 (3.9–26)	398
Vitamin E (mg)	7.9 (7.1–8.7)	2.7 (1.5–3.9)	34	5 (0.3–9.9)	65	6 (4.0–7.0)	70	4.4 (2.2–6.5)	55	8.7 (5.0–12)	110	6.2 (4.2–8.2)	78	16.2 (9.6–23)	205	12.2 (8.8–16)	153
*Vitamin K (µg)	118 (94–142)	88 (34–142)	75	112 (7–217)	95	112 (47–178)	95	68 (19–117)	57	90 (49–131)	76	107 (35–247)	90	139 (69–208)	117	143 (75–211)	121
Selenium (µg)	101 (96–106)	39 (27–52)	39	37 (17–56)	36	76 (65–88)	75	59 (37–80)	58	107 (88–126)	106	83 (47–119)	82	125 (104–146)	124	202 (89–314)	200
Zinc (mg)	9.8 (9.2–10.4)	5.4 (3.4–7.3)	55	7 (2.6–5.7)	42	9 (6.4–12)	91	5.6 (3.0–8.2)	57	10.6 (9.6–12)	108	9.6 (5.3–14)	98	16.3 (12–21)	167	19.9 (7.2–33)	203

Difference in reported energy and nutrient intake by AN subtype did not present as much separation as anticipated. Eddy *et al*. [6] suggest that crossover from restricting to binge purge behavior in AN is suggestive of phases in the course of the disease. This notion is supported by Peat *et al*. [7] who suggest there is generally progression from restrictor to binge purge AN to bulimia nervosa in a sizeable number of patients, although other cross-over patterns are seen as well. Furthermore, a recent study from this dataset comparing restricting *versus* binge purge AN revealed that the binge purge type tended to engage in more episodes of virtually all eating disorder behaviors, including restriction, suggesting that subtype distinction may reflect a dimension of severity as opposed to a true qualitative subtype differentiation [29].

Participants reporting high energy intakes in quartile 4 of each AN subtype have a substantial discrepancy between body weight and energy intake as their body weights do not reflect adequate or excessive calorie consumption. It is possible that they over-reported their intake. Schebendach *et al*. [8] suggest that weight restored AN patients tend to significantly over-report intake and that this bias is increased at higher intake levels. It is possible that some women with AN, particularly the binge purge subtype, engaged in various forms of purging behavior to compensate for eating or binge eating episodes. Similarly, physical activity may be elevated in AN, and may account for the discrepancies observed in quartile 4. Indeed, two studies using doubly labeled water to assess total energy expenditure in young women with AN and bulimia nervosa show that because of high levels of physical activity, they expend as much energy as their healthy weight counterparts despite decreased basal metabolic rate [30,31]. These data support the notion that AN participants have a failure to consume enough energy to maintain a healthy body weight no matter what their reported intake is, which may be an important element in the diagnosis of AN.

A primary limitation of our study is the use of three 24-hours DRs as representative of actual dietary intake. Although this is generally considered acceptable for healthy individuals, it may not be adequate to represent usual nutrient intake in patients with eating disorders. By comparing reported intake of the present group to the DRIs, we may be substantially overestimating adequacy of these women’s diets as these standards represent usual rather than optimal intake. In this study, we report nutrients from foods, not supplements, and may therefore not be reporting total intake. Additionally, the number of subjects in each cell is small and may, therefore, affect our outcomes.

Reported nutrient intake may not reflect the quantity of absorbed nutrients due to purging behaviors. The timing of and type of purging behaviors will affect the actual nutrients absorbed. We have no way to assess the bioavailability of consumed nutrients but can make the assumption that food and nutrient utilization is lower in this group than in healthy populations. 

Analysis of nutrient intake of reported diets does not provide any information on eating patterns or eating behaviors. The present analysis did not assess if there are differences in the patterns of food intake, such as meal timing, volume consumed per eating episode, or specific food avoidance behaviors of participants with restricting *versus* binge purge types of AN. Recent EMA studies have examined the relationship of several eating disorder behaviors and their precipitating factors [9,17].

## 5. Conclusions

Our data demonstrate that intake is below adequate for most nutrients in participants reporting reduced energy intake and approaching adequate in those reporting high intake. However, reported intake of participants in quartile 4 exceeds estimated energy needs, yet they maintain very low body weights. Recognizing that AN patients diagnosed with either subtype may display characteristics of both types (*i.e.*, restriction and binge purge behaviors) will allow for more individualized nutrition care plans. The goal of nutritional intervention in patients with AN is the attainment of healthy body weight and improved nutrient intake. 

Although nutrient intake assessment is subject to error of both over- and under-reporting, our results provide insight into the dietary intake of malnourished AN patients. Counseling of these patients should be directed to dietary improvements in total food consumption to increase energy intake with additional focus on nutritional gaps presented by evaluation of individual food intake. Although these data provide some insight into possible nutrient inadequacies, thorough assessment of dietary intake and physical activity patterns of patients with AN is required to design appropriate individualized medical nutrition therapy.
